# Cross-talk between endothelial and tumor cells via basic fibroblast growth factor and vascular endothelial growth factor signaling promotes lung cancer growth and angiogenesis

**DOI:** 10.3892/ol.2015.2881

**Published:** 2015-01-15

**Authors:** HENG DU, HUI SHI, DALI CHEN, YUBIN ZHOU, GUOWEI CHE

**Affiliations:** Department of Thoracic Surgery, West China Hospital, Sichuan University, Chengdu, Sichuan 610041, P.R. China

**Keywords:** human umbilical vein endothelial cells, human lung cancer A549 cell line, tumor microenvironment, vascular endothelial growth factor, basic fibroblast growth factor, glucose-regulated protein-78

## Abstract

The present study aimed to investigate the origin and potential mechanisms of angiogenesis in lung cancer cells. Normal endothelial cells (ECs) were isolated from human umbilical vein ECs (HUVECs) and cultured. The human lung cancer A549 cell line was also used. The cross-talk model between the HUVECs and the A549 cell line was constructed *in vitro* using a Millicell co-culture system. Cluster of differentiation (CD)31 and CD146 were selected as markers of the HUVECs. CD105 was used as a marker of activated blood vessel ECs in the tumor microenvironment and glucose-regulated protein-78 (GRP-78) was used as a biomarker of the A549 cells. The four markers were detected by immunofluorescence, and the mean optical density was calculated. The growth curves were constructed using the cell proliferation reagent, WST-1. The expression of vascular endothelial growth factor (VEGF) and basic fibroblast growth factor (bFGF) in the media was measured using an ELISA. The average proliferation rates of the co-cultured HUVECs and A549 cells were significantly higher than those observed in the control groups. The fluorescence intensity of CD105 expression in the co-cultured HUVECs was higher than that in the control group. The fluorescence intensity of GRP-78 in the co-cultured A549 cells was higher than that in the A549 cells cultured alone. The average expression levels of VEGF and bFGF in the co-cultured model were higher than in the control groups. Therefore, it was hypothesized that cancer cells may induce the differentiation of normal ECs into vascular ECs via the secretion of VEGF and bFGF. Furthermore, vascular ECs can affect the proliferation and differentiation of cancer cells.

## Introduction

The vascular niche is a major compartment of the tumor microenvironment, and therefore may have a role in the initiation, progression and metastasis of tumors (1,2). The abundant blood supply provided by the vascular niche enables physicians to use enhanced computed tomography (CT) during clinical examinations. Enhanced CT is often used in order to distinguish between malignant and benign lung lesions. The role of angiogenesis in the pathogenesis of lung cancer is well recognized. Endothelial cells (ECs) constitute the vast majority of vascular cells, however, the origin of vascular ECs in the tumor microenvironment remains controversial. It is believed that vascular ECs may be derived from normal ECs adjacent to the tumor, from progenitor ECs in the peripheral circulation or from undifferentiated cancer cells (3).

The interaction between cancer cells and ECs may underlie the process of angiogenesis within the tumor microenvironment. Tumor cells may directly or indirectly promote the phenotypic conversion of normal ECs (4). In addition, the ECs may be induced by tumor cells to partake in angiogenesis, which in turn affects the behavior of the tumor cells (5).

Cluster of differentiation (CD)31 and CD146 are unique markers of normal ECs (6,7). By contrast, CD105 is rarely expressed by normal ECs, but is strongly expressed by activated and rapidly proliferating ECs (8). The glucose-regulated protein-78 (GRP-78) is expressed by tumor cells, whose roles are drawing growing attention (9,10). Vascular endothelial growth factor (VEGF) and basic fibroblast growth factor (bFGF), and their corresponding receptors, are involved in the process of normal physiological angiogenesis in developing human and mouse embryos (11). However, the pivotal mechanism and signaling pathways involved in EC phenotype conversion and the cross-talk between lung cancer cells and ECs is yet to be elucidated.

The present study aimed to investigate the interaction between lung cancer cells and ECs, and to identify the potential role of VEGF and bFGF in the angiogenesis of lung cancer.

## Materials and methods

### Cell culture

The human lung adenocarcinoma A549 cell line was provided by the Biomedical Ultrasonic and Gynecological Oncology Laboratory, West China Second University Hospital (Sichuan, China). The cell line was cultured in RPMI 1640 medium containing 10% fetal bovine serum at 37°C in 5% CO_2_.

### Primary culture of human umbilical vein endothelial cells (HUVECs)

The primary culturing of the HUVECs was performed according to the methodology described by Baudin (12). The present study was approved by the Ethics Committee of the West China Hospital and written informed consent was obtained from all patients. Umbilical cords were donated from 5 women who gave birth naturally, in the Obstetrics and Gynecology Department of West China Second University Hospital. Informed consent was obtained from the patients. First, the HUVECs were isolated from the umbilical vein vascular wall. The umbilical cord, measuring 10–30 cm, was then washed using 1× phosphate-buffered saline (PBS) several times to remove the remnants of blood. Next, the umbilical cord was digested using 0.2% collagenase II at 37°C for 15 min. The cell suspension was then collected and centrifuged in a closed tube at 95 × g for 10 min. The supernatant was discarded carefully. Next, the cell pellets were suspended in 4 ml endothelial cell medium-2 (ECM-2). The cells were then dissociated by gentle aspiration and repulsing using a pipette. The samples were incubated at 37°C in 5% CO_2_. The medium was replaced every 24 h. Subsequent to primary culturing, the samples were passaged two to four times prior to use.

### Immunofluorescence (IF)

The HUVECs were seeded into 24-well plates at a density of ~10^4^ cells per well and cultured for two days in ECM. Next, the cells were washed and fixed in 4% paraformaldehyde at 4°C for 15 min, washed with 1× PBS, blocked with 5% bovine serum albumin for 30 min and then incubated with the primary antibody at room temperature for 60 min. The expression of CD31, CD146, and GRP-78 was detected using monoclonal rabbit anti-human antibodies (dilution, 1:100; ab180175, ab75769 and ab108615, respectively; Abcam, Cambridge, UK) CD105 was detected using a monoclonal mouse anti-human antibody (dilution, 1:100; ab11414, Abcam). The negative control samples were concurrently incubated with 1× PBS. The secondary antibodies were fluorescein isothiocyanate-conjugated goat anti-rabbit and goat anti-mouse (dilution, 1:50; ZF-0311 and ZF-0312, respectively; ZSGB-BIO, Beijing, China). The nuclei were stained using DAPI (dilution, 1:1,000). The samples were viewed under an Olympus BX51 fluorescence microscope (Olympus, Tokyo, Japan) and analyzed using Image-Pro Plus 6.0 software (IPP 6.0; Media Cybernetics Inc., Rockville, MD, USA).

### Co-culture of the HUVECs and A549 cell line

The co-culture of the HUVECs and A549 cells was performed using the Millicell co-culture system (Millipore, Billerica, MA, USA). In total, ~10^3^ HUVECs were inoculated in the upper chamber of the culture system (pore size, 4 μm), while the A549 cells were plated in the lower chamber. Subsequent to a 12-h incubation with Dulbecco’s modified Eagle’s medium (upper chamber, 0.2 ml; lower chamber, 1.25 ml), the old medium was discarded, and new ECM-2 was added prior to an eight-day culture period. The HUVECs were incubated alone in the upper chamber, and the A549 cells incubated in the lower chamber were used as a negative control.

The WST-1 assay (Roche, Basel, Switzerland), performed as previously described (12), was used to quantify the rate of proliferation. Briefly, for the HUVECs cultured alone, ~1,000 HUVECs were added to the upper well and ~1.25 ml ECM-2 (without any cells) was added to the lower well. For the A549 cells cultured alone, ~1,000 A549 cells were added to the lower well and 0.2 ml ECM-2 was added to the upper well (without any cells). For the co-culture assay, ~1,000 HUVECs were added to the upper well (the volume was ~0.2 ml) and ~1,000 A549 cells were added to the lower well (the volume was ~1.25 ml). For each well, 40 μl WST was added to each well according to the specification and the cells were incubated for 4 h. Next, 100-μl samples were obtained from each well and transferred to 96-well plates. The absorbance was measured by a Varioskan Flash microplate reader (Thermo Fisher Scientific, Waltham, MA, USA) at a wavelength of 440 nm. The assay was repeated three times and the mean absorbance was recorded. Optical density (OD) was used to compare the rate of proliferation.

### ELISA

For the collection of the conditioned media, serum-free DMEM was used in this assay. Next, the supernatant was harvested, centrifuged at 855 × g for 5 min and then stored at −80°C. The expression of VEGF and bFGF in the conditioned medium of the co-culture system and the single-culture groups was measured using an ELISA kit (Life Technologies, Carlsbad, CA, USA). The standard curves were constructed separately with the quantities of VEGF and bFGF selected as 800, 400, 200, 100, 50 or 25 ng/ml, and 25, 12.5, 6.25, 3.125, 1.5625 and 0.78125 ng/ml, respectively. The absorbance was measured at 450 nm.

### Statistical analysis

The purity of the HUVECs was quantified by counting the ratio of CD31- or CD146-positive cells. Five fields of vision (magnification, ×40) per well and a total of three wells were randomly selected for analysis. The intensities of CD105 and GRP-78 were analyzed using IPP 6.0. The mean OD (MOD) was defined as the ratio of the sum of the integrated OD and the sum of the area. The results are presented as the mean ± standard error of the mean. The independent sample t-test was used. P<0.05 was used to indicate a statistically significant difference.

## Results

### Isolation of HUVECs from the umbilical vein

**Figure 1:**
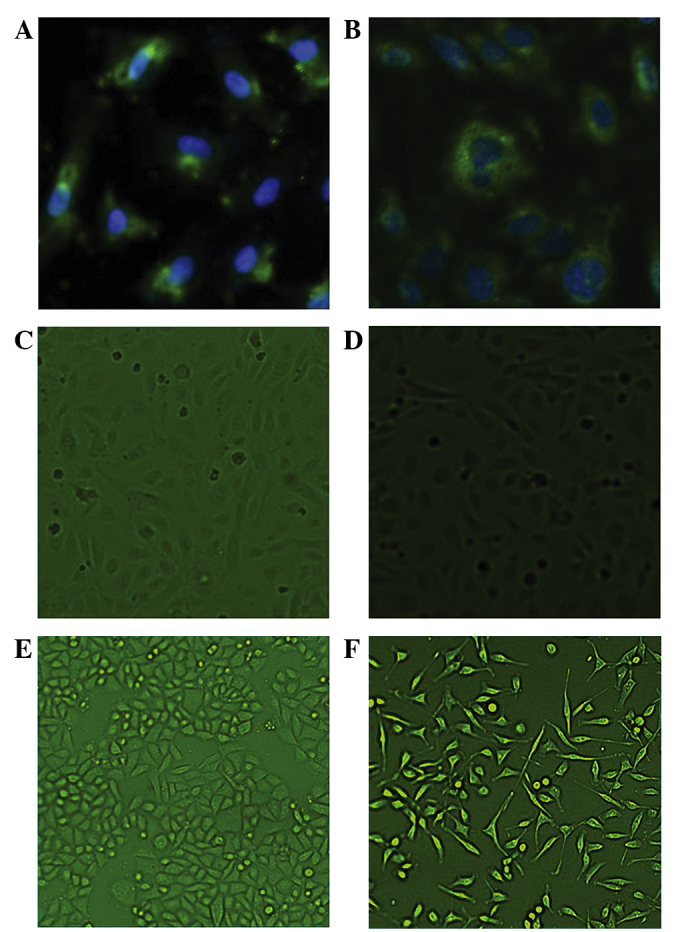
Immunofluorescence revealing the characteristics of the human umbilical vein endothelial cells (HUVECs) and the A549 cell line. (A) Cluster of differentiation (CD)31 and (B) CD146 expression in HUVECs (magnification, ×40). (C) The HUVECs that were cultured alone were teardrop-shaped (magnification, ×10) (D) The HUVECs in the co-culture group contained larger nuclei and elongated cell bodies, similar to the vascular ECs (magnification, ×10). (E) The A549 cells were fusiform or polygonal in shape when cultured alone (magnification, ×10). (F) In the co-culture system, however, these cells exhibited larger nuclei, with certain cells appearing binuclear or polynuclear. In addition, the cells became stretched and scattered (magnification, ×10).

The isolated cells were incubated with CD31 and CD146 antibodies and cultured according to the aforementioned method. The proteins were positively stained in the cytoplasm. The HUVECs demonstrated a high expression level of CD31 (range, 92.45–98.25%; mean, 96.15%) and CD146 (range, 96.20–98.00%; mean, 97.30%) compared with the control (Fig. 1A and B).

### Interaction between HUVECs and the A549 cell line

#### Morphological changes of the HUVECs and the A549 cell line

The HUVECs in the co-culture system exhibited a different morphology to those cultured alone. The HUVECs cultured alone were teardrop-shaped, with large nuclei and elongated cell bodies, similar in appearance to the vascular ECs cultured with the A549 cells (Fig. 1C and D).

The A549 cells were fusiform or polygonal in shape when cultured alone. In the co-culture system, however, the A549 cells exhibited relatively larger nuclei, with certain cells demonstrating binuclear or even polynuclear forms. Furthermore, the cells appeared stretched and scattered (Fig. 1E and F).

#### A549 cells promote the proliferation of HUVECs in the co-culture system

**Figure 2:**
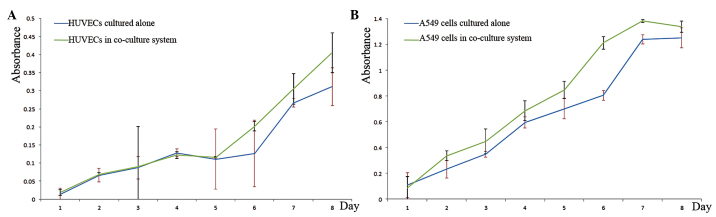
Proliferation of the human umbilical vein endothelial cells (HUVECs) and the A549 cell line. The proliferation rates of the HUVECs and the A549 cells were assayed using a WST-1 assay. The absorbance is presented as the mean ± standard error of the mean. (A) The proliferation rate of the HUVECs in the co-culture and culture-alone groups demonstrated no difference between days one and four (absorbance, 0.0741 and 0.0751, respectively; P=0.1845). However, the proliferation rate of the HUVECs in the co-culture group significantly increased on day five (absorbance, 0.8152 and 1.0274; P<0.001). On average during the eight days, the absorbance of the HUVECs in the co-cultured group was 0.1660±0.1304, while the absorbance of the control group cells was 0.1389±0.1003 (P=0.0448). (B) The mean absorbance of the A549 cells in the co-culture group was 0.7927±0.4877 compared with 0.6610±0.4304 in the cultured-alone group (P=0.036). It was therefore hypothesized that the cells interacted and promoted the proliferation of the other cell line.

The proliferation rate of the HUVECs was analyzed using the WST-1 assay, and the absorbance was measured. Compared with the control group, the proliferation of the HUVECs in the co-cultured system was significantly higher on day five (absorbance, 0.8152 vs. 1.0274; P<0.001). However, between days one and four there was no difference in terms of proliferation between the groups (absorbance, 0.0741 vs. 0.0751; P=0.1845). On average during the eight days, the absorbance of the HUVECs was 0.1660±0.1304 in the co-cultured system and 0.1389±0.1003 in the control group (P=0.0448) (Fig. 2A).

#### HUVECs promote the proliferation of A549 cells in the co-culture system

The proliferation of the A549 cell line was affected by the HUVECs. The mean absorbance of the co-culture group was significantly higher than that of the control group (0.7927±0.4877 vs. 0.6610±0.4304) (P=0.036)(Fig. 2B).

#### Phenotype conversion of HUVECs and the A549 cell line

**Figure 3:**
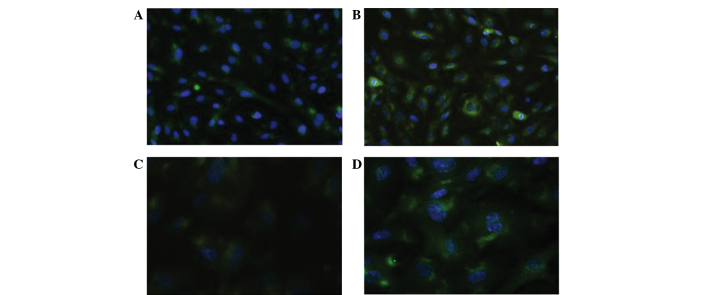
Immunofluorescence revealing the phenotype conversion of the human umbilical vein endothelial cells (HUVECs) and the A549 cells. Positive cytoplasmic expression of cluster of differentiation (CD)105 was observed in the HUVECs from (A) the negative control group and (B) the co-cultured group. A high expression level represented activated endothelium in the tumor microenvironment. Glucose-regulated protein-78 (GRP-78) was expressed in the cytoplasm of the A549 cells from (C) the control group and (D) the co-cultured group. The mean optical density of GRP-78 expression in the co-culture group and control group was 58.5987±7.1013 and 39.1734±2.2089, respectively. The co-culture group demonstrated a significantly increased expression of GRP-78 compared with the control group (P=0.0397).

CD105 is a unique marker of activated endothelial cells in the tumor microenvironment (8). The expression of CD105 in the HUVECs was detected using IF, and the MOD was calculated following eight days of co-culture. CD105 staining was positive in the cytoplasm of the HUVECs in the co-cultured system and in the negative control group. The MOD was significantly higher in the co-culture group than in the control group (40.3247±3.3343 vs. 23.2515±5.2713) (P=0.0027). The expression of CD105 was significantly higher in the co-culture group than in the control group. Furthermore, the effect of proliferation was downregulated (Fig. 3A).

GRP-78 is a molecular chaperone within the endoplasmic reticulum, and its expression is associated with the differentiation, invasion and drug-resistance of cancer cells (9,10). In the present study, the expression of GRP-78 was detected using IF, and the MOD was calculated. GRP-78 was expressed in the cytoplasm of the A549 cells. The MODs of the co-culture and control groups were 58.5987±7.1013 and 39.1734±2.2089, respectively. The expression of GRP-78 was significantly higher in the co-culture group than in the control group (P=0.0397), and the effect of proliferation was eliminated (Fig. 3B).

#### Contents of VEGF and bFGF in the medium

In the Millicell co-culture system, the HUVECs and A549 cells were unable to interact via direct contact, which suggests that cytokines may be involved in the cellular interactions. The expression levels of the cytokines, VEGF and bFGF, were measured in the conditioned medium of the co-cultured and control groups. The HUVECs that were cultured alone secreted extremely small amounts of VEGF and bFGF. The levels of VEGF were measured at 53.34±6.52 and 21.47±1.63 ng/ml (P<0.0001) and the levels of bFGF were 5.06±0.24 and 2.95±0.28 ng/ml (P<0.001) in the medium of the A549 single-cultured and co-culture groups, respectively.

In order to investigate the role of VEGF and bFGF without interference, HUVECs were cultured alone with serum-free DMEM. In total, 50 ng/ml VEGF or 1 ng/ml bFGF was artificially added to the medium, as recommended by Bai *et al* (13). The proliferation of the HUVECs was measured using a WST-1 assay. Compared with the HUVECs cultured in serum-free DMEM, the proliferation of the HUVECs in the VEGF(+) or bFGF(+) group was significantly higher (P<0.001). When the two factors were added consecutively, the effect upon HUVEC proliferation was significantly greater than that observed following the single addition of either factor alone (proliferation curves not shown).

## Discussion

Lung cancer is the leading cause of cancer-related mortality worldwide, and is therefore known for its high rates of morbidity and mortality. The highly progressive nature of the disease and its ability to metastasize make it incurable, and for any of its subtypes, the five-year survival rate is only ~15% (14). Overall, non-small cell lung cancer (NSCLC) accounts for 85% of all types of lung cancer (3). The rapid proliferation and metastatic nature of NSCLC cells relies upon support from tumor blood vessels in the form of angiogenesis (15). As tumor ECs (TECs) differ from normal ECs, tumor blood vessels demonstrate abnormal morphology. The interactions between TECs are aberrant, which leads to the formation of complex tumor blood vessels and uneven vessel diameters (16). In addition, TECs are unable to form normal monolayers, which leads to an incomplete barrier function of the tumor blood vessels and the occurrence of leakiness (17).

Due to the difficultly of isolating and culturing TECs from tumor tissues, few studies have focused on them. Furthermore, it has been suggested that the cells may lose their unique features following isolation. For these reasons, TECs are usually replaced by HUVECs. For a long time, TECs were considered to be phenotypically and cytogenetically normal. Following their successful isolation, it was realized that they differ from normal ECs in phenotype and express 46 unique tumor endothelial markers (18). In addition, TECs were identified to be karyotypically aneuploid, unlike normal ECs, which are diploid (19).

In the present study, the normal HUVECs expressed CD31 and CD146, which are two unique markers of normal ECs (20). The HUVECs exhibited a phenotype conversion when cultured with A549 cells. The phenotype of the co-cultured HUVECs became similar to that of the TECs, with a significant upregulation of CD105. CD105 (also known as endoglin) is an accessory protein belonging to the transforming growth factor-β receptor family, which is expressed in activated vascular ECs and has a key role in angiogenesis (7). The function of CD105 makes it important during embryonic development, and genetic mutations of this protein have been revealed to lead to Osler-Weber-Rendu syndrome (21). In solid tumors, the overexpression of CD105 is correlated with metastases and decreased survival (22).

Cancer cells can affect the phenotype and proliferation of TECs in a co-culture system, but TECs may also in turn affect tumor cells. The present study identified that A549 cells demonstrate morphological and phenotypic changes. When cultured with the HUVECs, the proliferation of the A549 cells increased. In addition, GRP-78 expression was detected in the A549 cells. GRP-78 was selected as a novel biomarker, as its level is associated with the differentiation, metastasis, chemoresistance and prognosis of tumor cells (10). Angiogenesis is known to promote tumor progression and metastasis by providing cells with the nutrients and oxygen necessary for growth and metastasis (23). The upregulation of GRP-78 in the A549 cells indicated that these cells were more prone to metastasis or progression to an advanced stage. This result coincided with that of a previous study (24). Despite this, the mechanism by which HUVECs affect A549 cells is yet to be elucidated.

Angiogenesis is a key process involved in physiological and pathological environments. A number of factors, including the key mediators, VEGF and FGF, take part in tumor angiogenesis (25). The present study demonstrated that HUVECs secrete extremely small amounts of VEGF. The expression of VEGF in the medium containing the A549 cells, however, was significantly higher than that observed in the co-culture group. Therefore, it was hypothesized that VEGF may act in a paracrine manner to affect the growth and proliferation of HUVECs. Under physiological conditions, angiogenesis is tightly regulated by a balance between anti- and pro-angiogenic factors. However, cancer cells are able to unbalance the related factors, and therefore promote angiogenesis. The VEGF secreted by cancer cells destroys the balance between anti- and pro-angiogenic factors, which promotes angiogenesis. VEGF is a homodimeric glycoprotein, which consists of two identical 23-kDa subunits. VEGF was first identified in the medium of an animal tumor model (26). VEGF is closely associated with lung diseases, such as pulmonary hypertension, acute respiratory distress syndrome, asthma and emphysema. In particular, high levels of VEGF have been identified in cases of lung adenocarcinoma (27). This association is highlighted by the fact that bevacizumab, a humanized monoclonal antibody against VEGF, is approved by the Food and Drug Administration to treat advanced NSCLC (25).

In addition to VEGF, FGF is a factor involved in cancer angiogenesis. FGF, which is a heparin-binding factor, can be divided into acidic FGF (aFGF) and basic FGF (bFGF) (28). bFGF can affect smooth muscle cells and ECs. Furthermore, it acts as a chemoattractant during the proliferation of ECs, which in turn promotes angiogenesis (29). The present study demonstrated that extremely small amounts of bFGF were secreted by the HUVECs, but that high levels were identified in the medium containing the A549 cells. In addition, only a small amount of bFGF was required for proliferation compared with VEGF (1 vs. 50 ng/ml). Therefore, it can be concluded that bFGF not only directly promotes HUVEC proliferation, but also acts indirectly by inducing the action of VEGF (28,29). In a previous study, bFGF was induced by hypoxia-inducible factor-α (29). As a result, the hypoxia induced an upregulation in the expression of bFGF in the tumor microenvironment, which promoted angiogenesis and resulted in the formation of a number of immature blood vessels. Due to the presence of immature blood vessels, cancer tissues are usually hypoxic (15). The hypoxic environment in turn promotes cancer cells to secrete more bFGF. Therefore, an antibody against bFGF may be more useful than one against VEGF. Although brivanib, a novel, orally available and selective receptor tyrosine kinase inhibitor that targets bFGF and VEGF receptors, is currently under clinical evaluation, further clinical trials are required (30).

Using a co-culture system, the present study examined the interplay between the A549 lung cancer cell line and tumor HUVECs. It was revealed that lung cancer cells may affect HUVECs in a paracrine manner. The secretion of VEGF and bFGF by cancer cells is found to potentially have key roles in promoting the proliferation of HUVECs. The A549 cells were also affected by the HUVECs at the same time. The upregulation of GRP-78 in cancer cells reflects that these tumor cells become more invasive and prone to metastasis. Angiogenesis is a very complicated mechanism and is likely to involve more factors. Additional research into angiogenesis and its potential underlying mechanisms is required.
